# Microstructure and Thermal Cyclic Behavior of FeNiCoAlTaB High-Entropy Alloy

**DOI:** 10.3390/ma18020387

**Published:** 2025-01-16

**Authors:** Li-Wei Tseng, Wei-Cheng Chen, Yi-Ting Hsu, Chih-Hsuan Chen

**Affiliations:** 1 Department of Mechatronics Engineering, National Changhua University of Education, Changhua 50007, Taiwan; earth520ya@gmail.com; 2 Department of Materials Science and Engineering, National Taiwan University, Taipei 10617, Taiwan; r09527035@ntu.edu.tw (Y.-T.H.); chchen23@ntu.edu.tw (C.-H.C.); 3 Department of Mechanical Engineering, National Taiwan University, Taipei 10617, Taiwan

**Keywords:** high entropy alloy, grain morphology, shape memory properties, rolling texture

## Abstract

This study investigates the grain morphology, microstructure, magnetic properties and shape memory properties of an Fe_41.265_Ni_28.2_Co_17_Al_11_Ta_2.5_B_0.04_ (at%) high-entropy alloy (HEA) cold-rolled to 98%. The EBSD results show that the texture intensities of the samples annealed at 1300 °C for 0.5 or 1 h are 2.45 and 2.82, respectively. This indicates that both samples were formed without any strong texture. The grain morphology results show that the grain size increased from 356.8 to 504.6 μm when the annealing time was increased from 0.5 to 1 h. The large grain size improved the recoverable strain due to a reduction in the grain constraint. As a result, annealing was carried out at 1300 °C/1 h for the remainder of the study. The hardness decreased at 24 h, then increased again at 48 h; this phenomenon was related to the austenite finish temperature. Thermo-magnetic analysis revealed that the austenite finish temperature increased when the samples were aged at 600 °C for between 12 and 24 h. When the aging time was prolonged to 48 h, the austenite finish temperature value decreased. X-ray diffraction (XRD) demonstrated that the peak of the precipitates emerged and intensified when the aging time was increased from 12 to 24 h at 600 °C. From the three-point bending shape memory test, the samples aged at 600 °C for 12 and 24 h had maximum recoverable strains of 2% and 3.6%, respectively. The stress–temperature slopes of the austenite finish temperature were 10.3 MPa/°C for 12 h and 6 MPa/°C for 24 h, respectively. Higher slope values correspond to lower recoverable strains.

## 1. Introduction

Conventional alloy design basically consists of selecting a main or principal element and then adding other elements to form the alloy. However, this traditional approach, which focuses on the terminal side of the phase diagram without exploring the vast central region, limits the potential for developing new alloys with improved properties. In 2004, Yeh et al. [1,2,3,4,5,6,7] developed the high-entropy alloy (HEA) design concept. An HEA was defined as having each of its principal element’s atomic percentages in the range of 5% to 35%. Subsequent research teams have expanded the compositional definition of high-entropy alloys to those alloys in which no single element exceeds 50% [8,9,10,11].

The Fe_40.5_Ni_28_Co_17_Al_11.5_Ta_2.5_B_0.05_ (at%) shape memory alloy (SMA), which exhibits a large recoverable strain of superelasticity (13.5%) and high tensile strength (1.2 GPa), was first reported by Tanaka et al. [12], who discovered that adding the tantalum (Ta) to the Fe-Ni-Co-Al-based SMA transformed its martensitic transformation from non-thermoelastic to thermoelastic. Additionally, adding a small amount of boron (B) is sufficient to inhibit the formation of a brittle second phase. Recent studies of the Fe-Ni-Co-Al-X and Fe-Ni-Co-Al-X-B-based (X: Ti, Nb, Ti + Nb and Ta) SMA systems have demonstrated a greater than 1.5% recoverable strain of superelasticity and shape memory effect in both polycrystalline and single-crystal structures [13,14,15,16,17,18,19,20,21,22,23]. The new Fe-Ni-Co-Al-X-based system is a promising candidate material for different applications, such as in dampers and actuators in the aerospace, robotic and automobile industries [24,25].

In 2019, Zhang et al. [26] designed the non-equiatomic Fe_43.76_Ni_27.5_Co_16.5_Al_10_Ta_2.2_B_0.04_ (at%) high-entropy alloy. The FeNiCoAlTaB HEA not only demonstrated high tensile strength but also superelastic behavior. When the alloy was aged at 700 °C for 5 h (h) under tensile stress at room temperature, the tensile strain reached 12%, with a maximum stress value of 1 GPa before alloy fracture. In a superelastic test, the maximum superelastic strain was around 2.5% at room temperature and 1% at a cryogenic temperature (−196 °C). For a compressive sample aged at 700 °C for 96 h, the maximum compressive stress value exhibited was around 1.4 GPa at room temperature. In the three-point bending test, a strain of around 1% was first applied to a sample in liquid nitrogen; the sample then recovered to its original shape after unloading. Based on uniaxial tensile test results, the FeNiCoAlTaB HEA showed high ductility in a wide temperature range between −196 °C and 800 °C. The ductility is 12% at −196 °C and 21% at 800 °C [27].

The FeNiCoAlTaB HEA single-crystal micro-pillars with <100>, <111> and <110> orientation were used to investigate the effect of orientation on superelasticity [28]. The alloy was cold-rolled to 90%. The annealing and aging heat treatment conditions for this cold-rolled alloy were 1300 °C-0.5 h and 700 °C-5 h, respectively. The single crystals with <100>, <111> and <110> orientation were cut from the aged samples. The bright filed transmission electron microscope images demonstrate the precipitate’s size was between 4 and 8 nm and the precipitate’s volume fraction was 28% for alloy after aging at 700 °C for 5 h. From the compressive stress–strain results, the recoverable strain along <001>, <011> and <111> is 2.8%, 0.5% and 1.2%, respectively. The recoverable strain is smaller than the theoretical values. The reason is related to the selection of martensite variants. Based on the theory, one variant is in the compression direction, and it will suppress the formation of the corresponding variant pair (CVP). As a result, the dislocations are generated due to the incompatibility between the austenite and martensite interface. The dislocations inhibit the martensite to reverse back to austenite and result in a small recoverable strain.

Huang et al. [29] investigated the compressive properties of an FeNiCoAlTaB high-entropy alloy using dynamic mechanical properties. After hot-rolling with a reduction ratio of 30% and aging at 600 °C for 24 h, the alloy had an average grain size of around 400 μm and a random texture. The size and volume fraction of the precipitates were 4 nm and 38%, respectively. Based on the compression true stress–true strain curves at three different strain rates, the yield strengths for strain rates of 10^−3^/s and 10^−2^/s were approximately 900 MPa and 950 MPa, respectively; there were no significant differences between the two strain rates. However, when the strain rate was 10^−3^/s, the yield strength was drastically increased to 1300 MPa. The high yield strength was attributed to stress-induced martensitic transformation and deformation-induced microbanding. The development of microbands leads to the development of thin-plate martensite and microbands.

Zhang et al. [26] performed a superelastic test at room temperature for both tension and compression. Moreover, a pure bending test was carried out without investigating the shape memory effect or properties via a thermal cyclic test. In addition, mechanical tests were conducted, focused on a 700 °C aging condition. Considering the previously conducted work, this study characterizes the shape memory properties of an FeNiCoAlTaB high-entropy alloy aged at 600 °C by using microstructural and thermal cyclic three-point bending tests.

## 2. Materials and Methods

**Figure 1:**
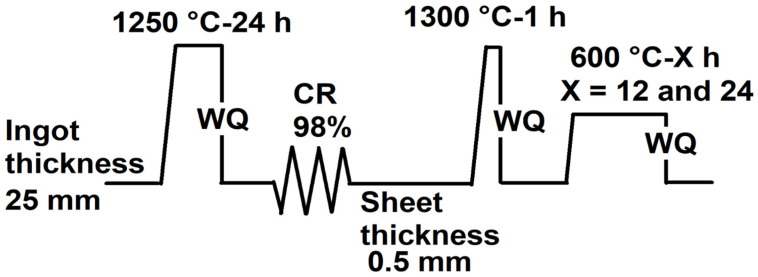
Illustration of the thermomechanical processes of NCATB-HEA.

The Fe_41.265_Ni_28.2_Co_17_Al_11_Ta_2.5_B_0.04_ (at%) high-entropy alloy (named NCATB-HEA) was synthesized via vacuum induction melting (VIM) and cast into an alloy bar. Wire electrical discharge machining (EDM) was used to cut the bar into several blocks, with each block having a length, width and thickness of 100 mm, 25 mm and 25 mm, respectively. The blocks were homogenized at 1250 °C for 24 h, followed by water quenching (WQ). The homogeneous bar was cold-rolled (CR) with a reduction ratio of 98% (CR98) to a thickness of 0.5 mm. Samples for the three-point bending test were cut from the 98% cold-rolled sheets with lengths, widths and thicknesses of 30 mm, 3 mm and 0.5 mm, respectively. Each test sample was annealed at 1300 °C for 1 h. The annealed samples were aged at 600 °C for 12 or 24 h (PXL600 °C-12 h and PXL600 °C-24 h). The three-point bending test device was a TA Q800 (TA Instruments, New Castle, DE, USA). The support span was 20 mm, and the cyclic temperature range was between 120 °C and −145 °C. Figure 1 presents the thermomechanical processing. The procedure is shown in the following:

The transformation temperatures of the NCATB-HEAs that had been aged at 600 °C for 12, 24 or 48 h were characterized using a superconducting quantum interference device (SQUID). Magnetic fields of 0.05 and 7 Tesla were applied, with a heating and cooling rate of 5 °C min^–1^. The test was conducted at −260 °C and 120 °C. The SQUID device used was an MPMS-3 (Quantum Design, San Diego, CA, USA).

The different phases or crystal structures under different thermomechanical processing conditions were measured via X-ray diffraction (XRD). The device used was a D5000 (Siemens, Aubrey, TX, USA). A range of 2 Thea was selected from 30 to 100 degrees. The microhardness after aging (600 °C for 3, 6, 12 h, 24 h or 48 h) was evaluated using a Vicker’s hardness testing machine. The hardness testing device used was an FM-310 (FUTURE-TECH CORP, Kawasaki, Japan). Optical microscopy (OM) was used to observe the microstructure of NCATB-HEA samples. Scanning electron microscopy (SEM) coupled with energy-dispersive spectrometry (EDS) was used to analyze the composition of the β phases and matrix in aging conditions.

To investigate the grain morphology of NCATB-HEA after different annealing times, the CR98 sample was annealed at 1300 °C for 0.5 and 1 h. The recrystallization texture and grain size of samples were obtained by electron backscatter diffraction (EBSD). The device was a JeoL JSM-7800F device (JEOL, Musashino, Akishima, Japan). The etching solution for preparing the EBSD samples was 90% C_2_H_5_OH and 10% HClO_4_. Inverse pole figures (IPFs) were used to measure the grain orientation distribution in three directions of samples: the rolling direction (RD), the normal direction (ND), and the transverse direction (TD). The orientation distribution functions (ODFs) results were plotted by selecting the {111}, {200} and {311} incomplete pole figure data. The phi2 is selected for 0, 45 and 65 degrees of the Euler space in FCC metals to understand the texture development in different thermomechanical processing. In the ODF results, f(g) is the orientation density, and f(g)_max_ is the maximum orientation density.

## 3. Results and Discussion

### 3.1. Evolution of Texture in NCATB-HEA

**Figure 2:**
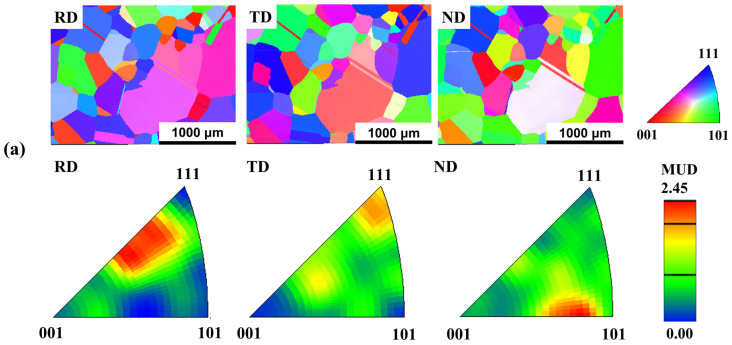

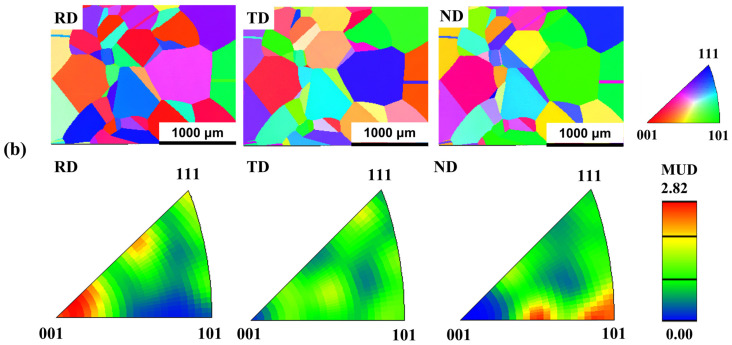
IPF maps of NCATB-HEA: (**a**) CR98 + 1300 °C, 0.5 h and (**b**) CR98 + 1300 °C, 1 h. RD, ND and TD represent the rolling direction, normal direction and transverse direction, respectively.

Figure 2a,b represent the EBSD inverse pole figure images along the rolling, transverse and normal directions in NCATB-HEA (CR98) samples that have been annealed at 1300 °C for 0.5 h and 1 h, respectively. Based on these results, the maximum texture intensity is 2.45 multiples of unity distribution (MUD) for 1300 °C-0.5 h and 2.82 MUD for 1300 °C-1 h. The results indicated that no strong recrystallization texture is formed when NCATB-HEA (CR98) is annealed at 1300 °C. Interestingly, the EBSD results are different from those reported by Zhang et al. [26]. In their experiments, a strong texture is developed in the <100> orientation for the cold-rolled sample annealed at 1300 °C for 0.5 h. One possible reason for this is the difference in thermomechanical processing between the two studies. In [26], the NCATB-HEA block is first hot-rolled at 1250 °C and then cold-rolled to 97.5% [26]. In our study, the NCATB-HEA block is first solution-heat-treated at 1250 °C for 24 h and quenched in water. The homogeneous bar is then directly cold-rolled with a thickness reduction of 98%.

**Figure 3:**
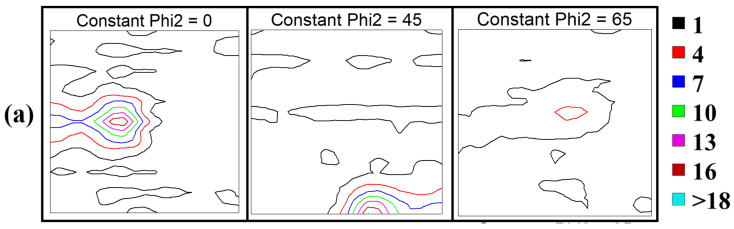

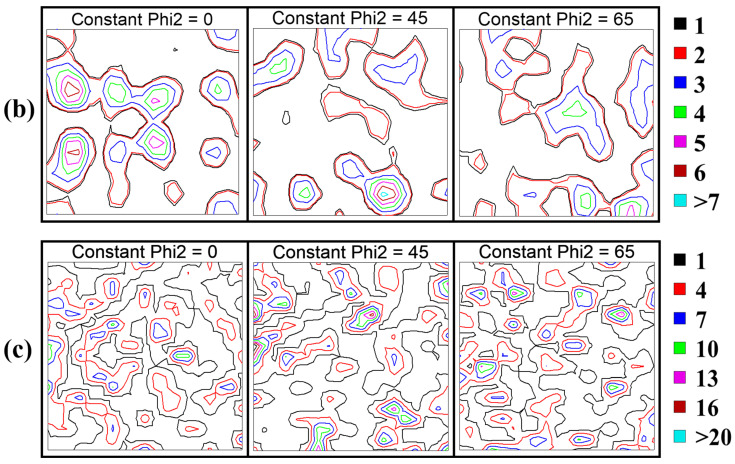
Orientation distribution function for phi2 = 0°, 45° and 65° in NCATB-HEA: (**a**) CR98, (**b**) CR98 + 1300 °C, 0.5 h and (**c**) CR98 + 1300 °C, 1 h.

Figure 3a–c shows the ODFs for CR98, CR98 + 1300 °C-0.5 h and CR98 + 1300 °C-1 h, respectively, at phi2 = 0°, 45° and 65°. The ODF can be used for the quantitative analysis of texture development during different thermomechanical processes. The CR98 sample showed Goss {011}<100> texture (f(g)_max_ = 8.98), Brass {011}<211> texture (f(g)_max_ = 18.51), G/B {110}<115> texture (f(g)_max_ = 8.2), S {123}<634> texture (f(g)_max_ = 5.32), Rt-Cu {112}<011> texture (f(g)_max_ = 2.15) and Cu {112}<111> texture (f(g)_max_ = 2.1). The CR98 sample has a strong Brass texture. The CR98 + 1300 °C-0.5 h sample showed Goss texture (f(g)_max_ = 3.38), Brass texture (f(g)max = 7.73), G/B texture (f(g)max = 6.96), A {110}<111> texture (f(g)_max_ = 5.85), Cu texture (f(g)_max_ = 2.81), Rt/G {011}<011> texture (f(g)_max_ = 3.36, S texture (f(g)_max_ = 4.04) and Rt-Cu texture (f(g)max = 2.2). The CR98 + 1300 °C-1 h sample showed Goss texture (f(g)_max_ = 12.88), Brass texture (f(g)_max_ = 10.19), G/B texture (f(g)_max_ = 10.19), A texture (f(g)_max_ = 11.61), Cu texture (f(g)_max_ = 8.29), Rt/G texture (f(g)_max_ = 4), S texture (f(g)_max_ = 9.6). RT-C {001}<110> texture (f(g)_max_ = 8.69) and Rt-Cu texture (f(g)_max_ = 22.33). From the preceding ODF results, we conclude the main textures for the CR98 sample are Goss, Brass and G/B. After annealing, the f(g)_max_ values for these three textures decrease, and different textures are formed. When the annealing time is increased from 0.5 h to 1 h, the f(g)_max_ value of the Rt-Cu texture increases. The major texture components in this instance are Rt-Cu, Goss, Brass and G/B.

### 3.2. Grain Morphology in NCATB-HEA

**Figure 4:**
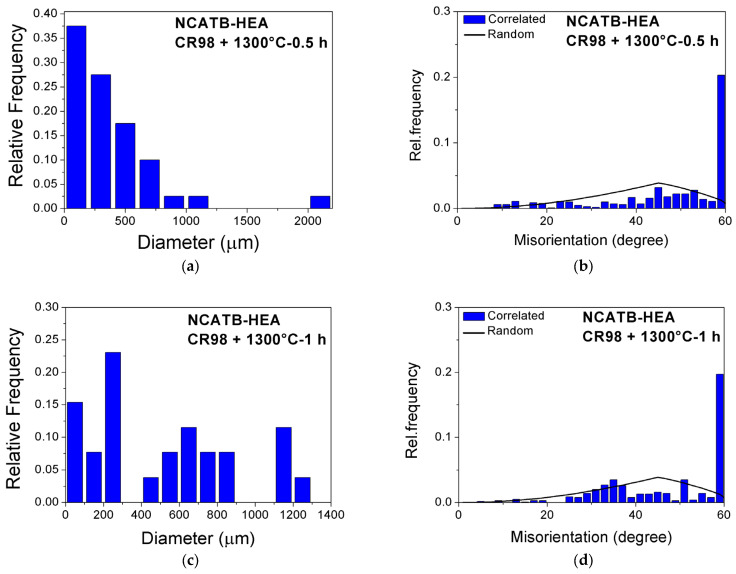
Grain diameter distribution and the misorientation distribution for NCATB-HEA: (**a**,**b**) CR98 + 1300 °C, 0.5 h and (**c**,**d**) CR98 + 1300 °C, 1 h.

In Fe-based SMA systems, enlarging the grain size not only decreases the number of constrained grains but also improves the recoverable strain. Based on the results from Tanaka et al. and Zhang et al. [12,26], the average grain size in the Fe-Ni-Co-Al-based system should be above 400 μm. In this study, the grain size distributions for NCATB-HEA after 98% CR when annealing at 1300 °C for 0.5 and 1 h are shown in Figure 4a,c. The average grain sizes in the samples after annealing at 1300 °C for 0.5 and 1 h are 356.8 and 504.6 μm, respectively. The results show that a large grain size can be obtained when annealing at 1300 °C for 1 h. Therefore, this annealing condition was selected for use in the hardness, SQUID, XRD, and three-point bending measurements. In addition, brittle second phases (β-NiAl) will be formed at the grain boundaries or triple junctions during the aging process. These brittle second phases are more likely to form at high-angle grain boundaries (HABs), where misorientation is greater than about 15 degrees. In contrast, low-angle grain boundaries (LABs) represent a misorientation of less than about 15 degrees. LABs are thought to reduce the generation of brittle second phases (β-NiAl). Figure 4b,d display the misorientation distribution after annealing at 1300 °C for 0.5 h and 1 h. The percentage of LABs was 5% and 3% for the 1300 °C-0.5 h and 1300 °C-1 h samples, respectively. For the FeNiCoAlTaB HEA, the LAB fraction can reach 60%; the percentage of LABs in Zhang’s report was about 25% [26]. The differences in the LAB percentages may be related to differences in the thermomechanical processing, but further study is required.

### 3.3. Crystal Structure in NCATB-HEA

**Figure 5:**
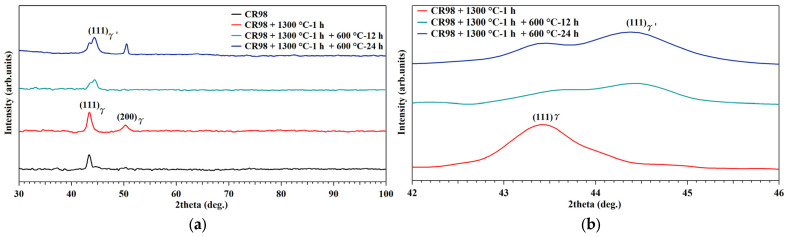
XRD results for NCATB-HEA samples under various thermomechanical processing conditions: 2 theta (**a**) between 30 and 100 degrees and (**b**) 42 and 46 degrees.

The results from X-ray diffraction measurements of the NCATB-HEA CR98 samples under various thermomechanical processing conditions are shown in Figure 5a. The CR98 sample exhibits a diffraction peak corresponding to the (111) diffraction plane of the austenite phase (γ) with an FCC structure. The sample (annealed at 1300 °C for 1 h) shows diffraction peaks in both the (111) and (200) planes of the austenite phase. For aged samples (PXL600 °C-12 h and PXL600 °C-24 h), a new diffraction peak with a (111) diffraction plane of the precipitate (γ′) phase with an L1_2_ structure emerged. The intensity of the new peak increased when the aging time was increased from 12 to 24 h, as shown in Figure 5b. This indicates that the increasing intensity of the precipitate peak reflects the increase in the volume fraction of the precipitates. A similar XRD result observation was reported by Zhou et al. [30]. In addition, the study by Fu et al. [18] indicated that the brittle second phases (β-NiAl) appear at around 65 degrees for FeNiCoAlNbB shape memory alloys. In the present study, a diffraction peak at 65 degrees was not detected in the PXL600 °C-12 h and PXL600 °C-24 h samples. This indicates that a small number of second phases are generated under these aging conditions. The same observation was reported by Zhang et al. [23]. They found that no obvious β phase XRD peak (around 65 degrees) is observed in NCATB-HEA samples due to the small amount of β phase. In order to further investigate the small amount of β phases, the OM and SEM were used to characterize the microstructure of NCATB-HEA samples.

### 3.4. Microstructure in NCATB-HEA

**Figure 6:**
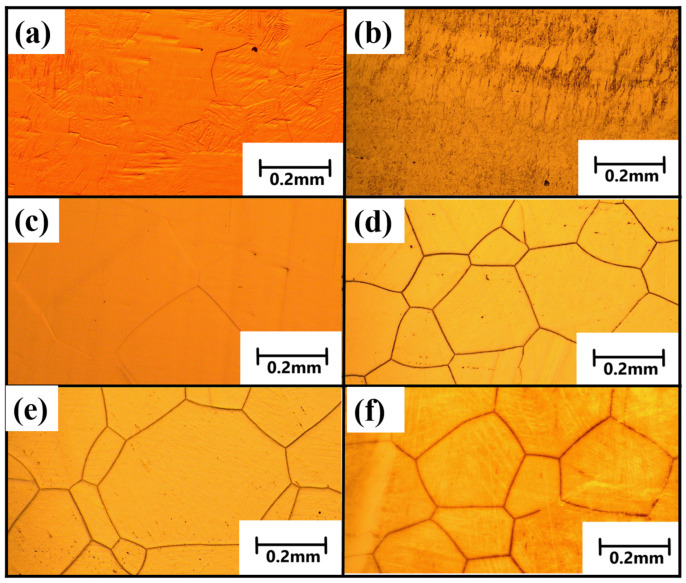
Microstructure of NCATB-HEA samples in different thermomechanical processing conditions: (**a**) as-received, (**b**) CR98, (**c**) CR98 + 1300 °C-1 h, (**d**) CR98 + 1300 °C-1 h + 600 °C-12 h, (**e**) CR98 + 1300 °C-1 h + 600 °C-24 h and (**f**) CR98 + 1300 °C-1 h + 600 °C-48 h.

Figure 6a–f show the optical microscope of the NCATB-HEA in different thermomechanical processing conditions. From OM results, the CR98 sample shows blurred grain boundaries due to the large percentage of rolling reduction. Figure 6c shows the CR98 sample annealed and heat-treated at 1300 °C for 1 h. The grain boundaries are clearly observed in this condition. Figure 6d–f display the CR98 sample after annealing at 1300 °C for 1 h and aged heat treatment at 600 °C for 12, 24 and 48 h. From the microstructure results, β phases are not clearly observed for the 600 °C-12 h and 600 °C-24 h samples, and observable β phases are generated along the grain boundary until the superconducting quantum interference device aging time is increased to 48 h. Because the width of the β phases is thick compared to another alloy’s system, it is difficult to identify the β phases at the grain boundary, especially for the 600 °C-12 h and 600 °C-24 h samples. In order to further observe β phases, the SEM was carried out in three aged samples.

**Figure 7:**
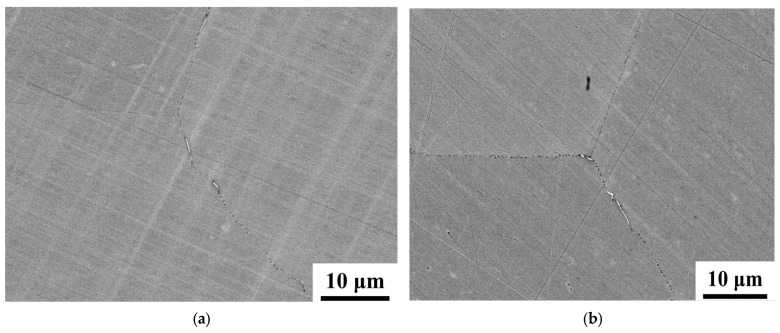

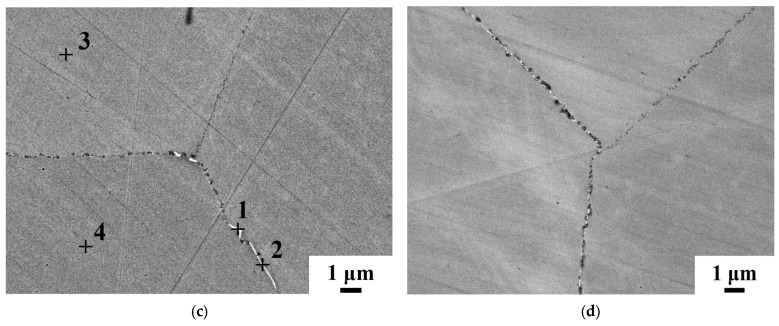
Backscattered electron micrograph of the NCATB-HEA aged samples: (**a**) 600 °C-12 h, (**b**) 600 °C-24 h, (**d**) 600 °C-48 h, and (**c**) high magnification at triple junction for 600 °C-24 h aged sample.

Figure 7a–d show the backscattered electron (BSE) image of NCATB-HEA samples under various aging conditions. Figure 7a,b present the BSE images of the 600 °C-12 h and 600 °C-24 h samples. Because the aging time is not long enough to form a large amount of β phases, only a few β phases (bright clusters) are formed along the grain boundary. When the aging time is increased to 48 h, increasing numerous β phases are formed along the grain boundaries (Figure 7d). Figure 7c shows the high magnification of the BSE image for 600 °C-24 h samples. The numbers in a figure indicate the EDS points. Numbers 1 and 2 are analyses in β phases, and Numbers 3 and 4 are analyses in matrices. The EDS result is summarized in Table 1 for the 600 °C-24 h samples. Table 2 summarizes the EDS results of the matrix for the 600 °C-12 h and 600 °C-48 h samples. Based on EDS results, the β phases (white clusters) contain high Ta content compared to the matrix. Zhang et al. [23] have found that Ta element first precipitates in the triple-junction area and then accumulates along the high-angle grain boundaries during the aging process. β phases prefer to nucleate and grow from Ta-rich regions. In this study, the width of β phase for three aging samples is less than 1 μm. Based on the OM and SEM results, the small amount of β phases generated in this alloy is due to three reasons. First, the addition of boron is effective in suppressing Ta formation at the grain boundary. Ta first precipitates along the grain boundaries, and β phases prefer to nucleate from Ta regions. If the Ta regions are decreased, the amount of β phases is small. Boron will retard the generation speed of the second phases during the aging process and results in decreasing the thickness of the β phase along grain boundaries. Moreover, the aging time is shorter than 72 h. Therefore, the aging time is not long enough to form a large amount of β phases. Additionally, grain boundary (GB) segregation can alter the mechanical properties. Tanaka et al. [31] reported that β phases reduce the yield strength of the HEA due to the facility of GB sliding. The AlCoCrFeNi HEA with Al atom segregation at GB demonstrates the GB stabilization effect; the formed elemental GB segregation of Al decreases the amount of β phase, leads to resistance to the GB sliding, and improves the yield strength.

### 3.5. Hardness in NCATB-HEA

**Figure 8:**
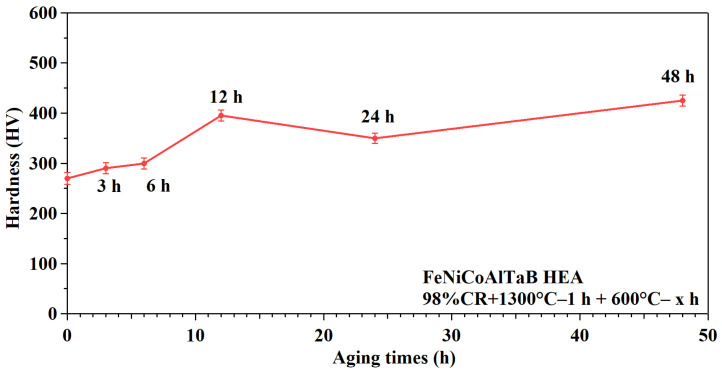
Hardness of an NCATB-HEA CR98 sample under various aging conditions.

Figure 8 and Table 3 represent the hardness value measurements in NCATB-HEA CR98 samples under various aging conditions. The hardness for CR98 + 1300 °C, 1 h is 270 HV. After aging at 600 °C for 3, 6, 12, 24 and 48 h, the hardness values are 290 HV, 300 HV, 395 HV, 350 HV and 425 HV, respectively. The hardness increases when the aging time is increased from 3 to 12 h. However, the hardness decreases as the aging time is increased from 12 to 24 h. The hardness increases again when the aging time is prolonged from 24 to 48 h. The hardness value is lower at 48 h than at 12 h. The reason for this is related to the transformation temperature. Consequently, SQUID measurements were performed on samples aged for 12, 24 and 48 h.

### 3.6. Transformation Temperatures in NCATB-HEA

**Figure 9:**
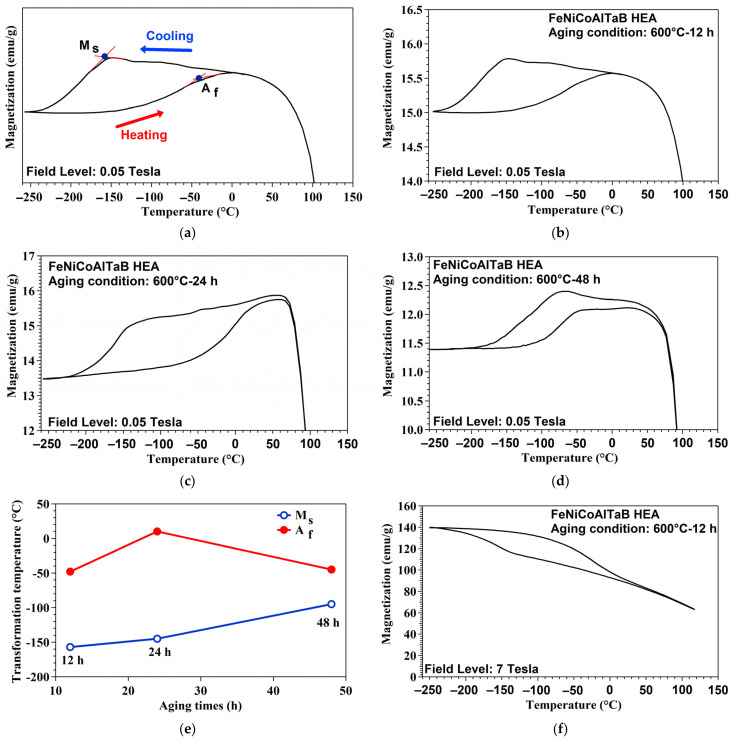

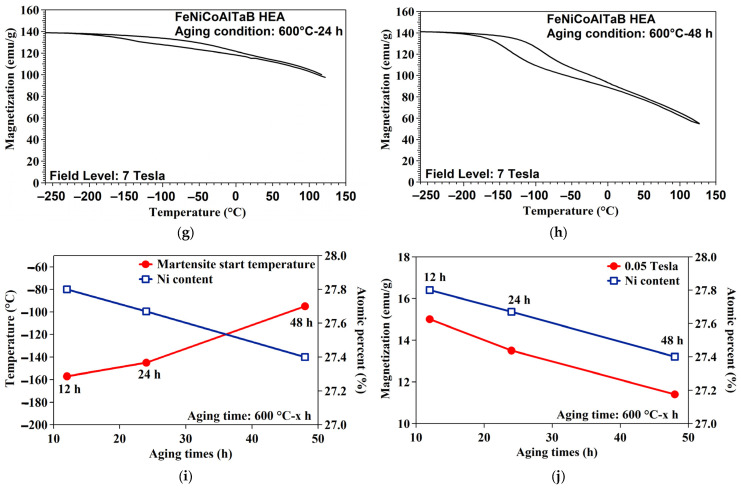
Magnetization–temperature (M-T) curves measured at 0.05 Tesla and 7 Tesla for NCATB-HEA under different aging conditions. (**a**) Demonstration of how transformation temperatures are determined via thermo-magnetic test. Field level: 0.05 Tesla for (**b**) 600 °C for 12 h, (**c**) 600 °C for 24 h and (**d**) 600 °C for 48 h. (**e**) The relationship between transformation temperatures (A_f_ and M_s_) and aging time. Field level: 7 Tesla for (**f**) 600 °C for 12 h, (**g**) 600 °C for 24 h and (**h**) 600 °C for 48 h. (**i**) Martensitic start temperatures and Ni content as a function of aging time. (**j**) Magnetization and Ni content as a function of aging time.

The transformation temperatures, such as the austenite finish temperature (A_f_) and martensite start temperature (M_s_), of NCATB-HEA under different aging conditions were characterized using superconducting quantum interference device (SQUID). The transformation temperatures are shown in Figure 9a. Figure 9b–d present the magnetization vs. temperatures measured at 0.05 Tesla for NCATB-HEA aged at 600 °C for 12, 24 and 48 h. Based on this measurement, the A_f_ and M_s_ values were −48 °C and −157 °C for the PXL600 °C-12 h sample, 10 °C and −145 °C for the PXL600 °C-24 h sample and −55 °C and −95 °C for the PXL600 °C-48 h sample. The relationships between the transformation temperatures (A_f_ and M_s_) and the three aging conditions are plotted in Figure 9e. The results show that the transformation temperature increases as the aging time increases from 12 to 24 h. However, the transformation temperature tends to decrease as the aging time is prolonged from 24 to 48 h. Since increasing A_f_ should render stress-induced martensitic transformation easier at room temperature, hardness is expected to decrease as the aging duration increases. These results are in line with the results from our hardness experiments, which show that the values start to decrease from 12 to 24 h and increase from 24 to 48 h. The results for aged samples of NCATB-HEA in a strong magnetic field (7 Tesla) are shown in Figure 9f–h. The M_s_ and A_f_ values of NCATB-HEA are presented in Table 4. The transformation temperatures (A_f_ and M_s_) of the three aging conditions increase as the magnetic field is increased from 0.05 to 7 Tesla, i.e., increasing the strength of the magnetic field increases the transformation temperature.

From the previous EDS results of the matrix for NCATB-HEAs that had been aged at 600 °C for 12, 24 and 48 h, the Ni concentration decreases with increasing aging times. Figure 9i summarizes the effect of aging treatment on the martensite start temperature (determined from 0.05 Tesla) and Ni content in three aging conditions. From the results, the Ni content decreases, and the martensite start temperature increases with increasing aging times. This indicates that decreasing the Ni content leads to an increase in the martensite start temperature [32]. Figure 9j shows the relation between magnification (determined from 0.05 Tesla) and Ni content. The results show that magnetization decreases with increasing aging times. As aging time increases, magnetization and Ni content both decrease.

### 3.7. Shape Memory Properties in NCATB-HEA

**Figure 10:**
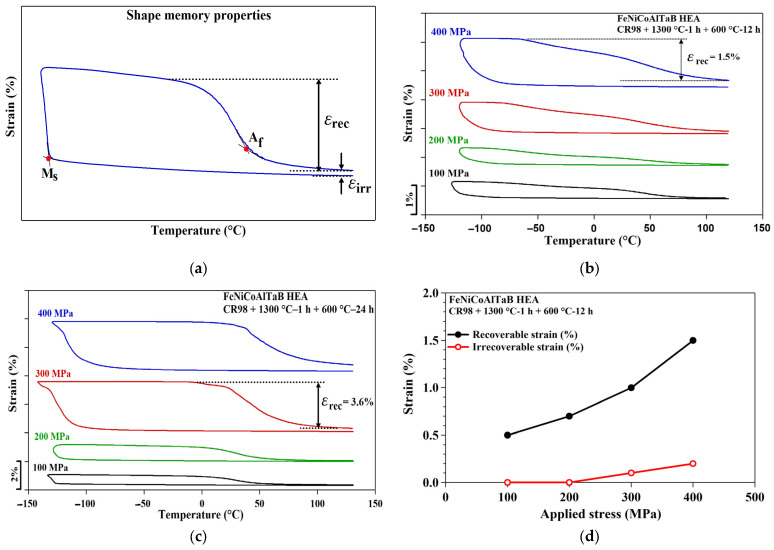

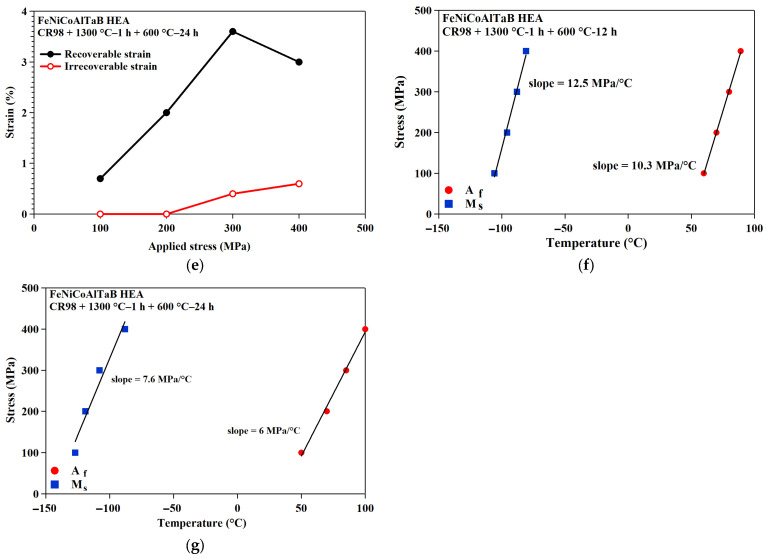
Thermal cyclic curves for the NCATB-HEA. (**a**) Illustration of shape memory properties: recoverable strain (ε_rec_), irrecoverable strain (ε_irrec_) and transformation temperatures (M_s_ and A_f_); strain vs. temperature curves measured using the three-point bending thermal cyclic test under different stress levels for (**b**) PXL600 °C-12 h and (**c**) PXL600 °C-24 h; (**d**,**e**) ε_rec_ and ε_irrec_ for both the PXL600 °C-12 h and PXL600 °C-24 h samples; (**f**,**g**) the relationship between the applied stress and transformation temperature for both the PXL600 °C-12 h and PXL600 °C-24 h samples.

The determination of shape memory properties, such as the recoverable strain (ε_rec_), irrecoverable strain (ε_irrec_) and transformation temperatures (M_s_ and A_f_), of the NCATB-HEA in the three-point bending test are illustrated in Figure 10a. Figure 10b,c show three-point bending thermal cyclic tests under different constant stresses for the PXL600 °C-12 h and PXL600 °C-24 h samples, respectively. Recoverable and irrecoverable strains are plotted as the functions of applied stress in Figure 10d,e for the PXL600 °C-12 h and PXL600 °C-24 h samples, respectively. Based on these results, the maximum recoverable strain was 1.5% for PXL600 °C-12 h and 3.6% for PXL600 °C-24 h. The applied stress dependence of the transformation temperatures taken from Figure 10b,c are summarized in Figure 10f,g. The relationship between the applied stress and the transformation temperature follows the Clausius–Clapeyron relation, as follows:

(1) $$\frac{d\sigma }{dT}=-\frac{\Delta H}{\varepsilon_{tr}.T_{0}}$$

where dσ/dT represents the stress–temperature slope, ΔH is the change in transformation enthalpy, T_0_ is the equipment temperature and ε_tr_ is the transformation strain or recoverable strain. From Figure 10f,g, stress–temperature slopes of M_s_ and A_f_ are 12.5 MPa/°C and 10.3 MPa/°C for PXL600 °C-12 h, respectively. For the PXL600 °C-24 h sample, the stress–temperature slope of M_s_ is 7.6 MPa/°C and that of A_f_ is 6 MPa/°C. The dσ/dT is inversely proportional to the ε_tr_. A larger slope corresponds to a smaller recoverable strain. The recoverable strain of the PXL600 °C-24 h sample is larger than that of the PXL600 °C-12 h sample. Therefore, the steep stress–temperature slope in PXL600 °C-12 h corresponds to a small recoverable strain. In another HEA system, the M_s_ and A_f_ slopes for a TiHfZrCuNi HEA are 10.12 and 13.20 MPa/°C, respectively [33].

## 4. Conclusions

This study investigated the grain morphology, hardness, magnetic responses and shape memory responses of cold-rolled samples of an FeNiCoAlTaB high-entropy alloy. The research results are as follows:

1. EBSD revealed that the average grain size was 356.8 and 504.6 μm for 1300 °C-0.5 h and 1300 °C-1 h, respectively. Both annealing conditions showed that no strong recrystallization texture is formed in NCATB-HEA (CR98).
2. The ODF results indicate that the textural components of the cold-rolled sample are Brass, Goss and G/B. The major texture components in the sample annealed at 1300 °C for 1 h were Rt-Cu, Goss, Brass and G/B.
3. The hardness value decreases from 12 to 24 h and increases from 24 to 48 h, which is related to the transformation temperatures. The transformation temperatures increase when the aging time increases from 12 to 24 h. However, the transformation temperature tends to decrease as the aging time is prolonged from 24 to 48 h. The SQUID results match with our hardness results.
4. XRD analysis revealed that a new peak in the (111) plane of the precipitates emerged, and its intensity increased when the aging time at 600 °C was increased from 12 to 24 h.
5. The three-point bending thermal cyclic results show that samples aged at 600 °C for 12 and 24 h showed maximum recoverable strains of 2% and 3.6%, respectively. The stress–temperature slopes for the austenite finish temperatures were 10.3 MPa/°C for 12 h and 6 MPa/°C for 24 h, respectively. The slope value for PXL600 °C-12 h is higher than for PXL600 °C-24 h. The reason for this is that the higher slope value corresponds to a lower recoverable strain.

## Figures and Tables

**Table 1:** The composition measurements of the 600 °C-24 h sample.

Element	β Phases			Matrix		
	1	2	Average	3	4	Average
Fe (at%)	43.2	42.3	42.86 * ± 0.5	43.9	43.1	43.57 * ± 0.2
Ni (at%)	26.3	27.4	26.73 * ± 0.6	27.2	28.2	27.67 * ± 0.2
Co (at%)	16.1	16.6	16.33 * ± 0.3	17.2	17	17.07 * ± 0.4
Al (at%)	8.5	9.2	8.8 * ± 0.4	9.5	9.4	9.5 * ± 0.1
Ta (at%)	5.9	4.6	5.3 * ± 0.7	2.2	2.3	2.2 * ± 0.1

* The standard deviations were calculated from 3 overall measurement points.

**Table 2:** The composition measurements of the 600 °C-12 h and 600 °C-48 h samples.

Element	Matrix (600 °C-12 h)			Matrix (600 °C-48 h)		
	1	2	Average	3	4	Average
Fe (at%)	44.2	42.9	43.63 * ± 0.6	44.3	43.3	43.76 * ± 0.5
Ni (at%)	27.5	27.9	27.8 * ± 0.3	27.2	27.7	27.4 * ± 0.3
Co (at%)	16.6	17.6	16.93 * ± 0.5	16.7	17.3	17.13 * ± 0.4
Al (at%)	9.3	9.4	9.33 * ± 0.6	9.5	9.4	9.4 * ± 0.1
Ta (at%)	2.3	2.2	2.26 * ± 0.5	2.3	2.3	2.3 * ± 0.1

* The standard deviations were calculated from 3 overall measurement points.

**Table 3:** Hardness results for the NCATB-HEA CR98 sample.

Thermomechanical Processing	Hardness (HV)
CR98 + 1300 °C-1 h	270 ± 12
CR98 + 1300 °C-1 h + 600 °C-3 h	290 ± 11
CR98 + 1300 °C-1 h + 600 °C-6 h	300 ± 11
CR98 + 1300 °C-1 h + 600 °C-12 h	395 ± 11
CR98 + 1300 °C-1 h + 600 °C-24 h	350 ± 10
CR98 + 1300 °C-1 h + 600 °C-48 h	425 ± 11

**Table 4:** M_s_ and A_f_ of NCATB-HEA.

Aging Condition	Magnetic Field	Transformation Temperature
600 °C-12 h	0.05 T	A_f_ = −48 °C and M_s_ = −157 °C
600 °C-12 h	7 T	A_f_ = −45 °C and M_s_ = −150 °C
600 °C-24 h	0.05 T	A_f_ = 10 °C and M_s_ = −145 °C
600 °C-24 h	7 T	A_f_ = 8 °C and M_s_ = −125 °C
600 °C-48 h	0.05 T	A_f_ = −55 °C and M_s_ = −95 °C
600 °C-48 h	7 T	A_f_ = −45 °C and M_s_ = −92 °C

## Data Availability

The original contributions presented in this study are included in the article. Further inquiries can be directed to the corresponding author.
